# *CHEK2 *contribution to hereditary breast cancer in non-*BRCA *families

**DOI:** 10.1186/bcr3062

**Published:** 2011-11-24

**Authors:** Alexis Desrichard, Yannick Bidet, Nancy Uhrhammer, Yves-Jean Bignon

**Affiliations:** 1 Laboratoire Diagnostic Génétique et Moléculaire, Centre Jean Perrin, 58 rue Montalembert, F-63011 Clermont-Ferrand, France; 2 Clermont Université, Université d'Auvergne, 28 place Henri Dunant, EA 4233, BP 10448, F-63001 Clermont Ferrand, France

## Abstract

**Background:**

Mutations in the *BRCA1 *and *BRCA2 *genes are responsible for only a part of hereditary breast cancer (HBC). The origins of "non-*BRCA*" HBC in families may be attributed in part to rare mutations in genes conferring moderate risk, such as *CHEK2*, which encodes for an upstream regulator of BRCA1. Previous studies have demonstrated an association between *CHEK2 *founder mutations and non-*BRCA *HBC. However, very few data on the entire coding sequence of this gene are available.

**Methods:**

We investigated the contribution of *CHEK2 *mutations to non-*BRCA *HBC by direct sequencing of its whole coding sequence in 507 non-*BRCA *HBC cases and 513 controls.

**Results:**

We observed 16 mutations in cases and 4 in controls, including 9 missense variants of uncertain consequence. Using both *in silico *tools and an *in vitro *kinase activity test, the majority of the variants were found likely to be deleterious for protein function. One variant present in both cases and controls was proposed to be neutral. Removing this variant from the pool of potentially deleterious variants gave a mutation frequency of 1.48% for cases and 0.29% for controls (*P *= 0.0040). The odds ratio of breast cancer in the presence of a deleterious *CHEK2 *mutation was 5.18.

**Conclusions:**

Our work indicates that a variety of deleterious *CHEK2 *alleles make an appreciable contribution to breast cancer susceptibility, and their identification could help in the clinical management of patients carrying a *CHEK2 *mutation.

## Introduction

Breast cancer is one of the main causes of cancer-related deaths among women worldwide, with 5% to 10% of cases being due to hereditary risk. However, mutations in the two major genes, *BRCA1 *and *BRCA2*, are found in only 15% to 20% of hereditary breast cancer (HBC) families [1]. Several studies have reported evidence that germline mutations in other susceptibility genes, such as *ATM, PABL2, BRIP1 *and *CHEK2*, might be the predisposing factor in some HBC families [2-5]. In addition, the lower penetrance of these mutations suggests that they might act in concert with other hereditary factors [6-10].

*CHEK2 *is the human homolog of *Rad53 *(*Saccharomyces cerevisiae*) and *Cds1 *(*Schizosaccharomyces pombe*). This family of kinases is characterized by several domains: a SQ/TQ cluster domain, a Forkhead-associated (FHA) domain and a Ser/Thr kinase domain [11]. In response to DNA double-strand breaks or replicative stress, CHEK2 is activated by the kinases ATM and ATR [12]. These proteins catalyze the phosphorylation of threonine 68 of CHEK2, causing its transient dimerization via the FHA domain. This leads to CHEK2 *trans*-autophosphorylation and its full activation [13]. Activated CHEK2 monomers phosphorylate, in turn, numerous downstream substrates, including the P53 tumor suppressor, CDC25 family proteins and serine 988 of BRCA1, activating cell-cycle checkpoints and increasing DNA repair efficiency [14-17]. These interactions suggest that CHEK2 may also play a role in breast cancer [14].

Germline *CHEK2 *mutations are associated with breast cancer in different populations. For example, heterozygosity for the well-studied c.1100delC mutation, present in 1.4% of the Finnish population and in 0.2% of the Polish population, confers a relative risk for developing breast tumors of about 2 for women and 10 for men [18,19] Likewise, the variant Ile157Thr, present in 5.3% of the Finnish population and in 4.8% of the Polish population, confers a relative risk of breast cancer of 1.5 [20,21].

However, very few groups have studied the entire *CHEK2 *gene in HBC [22-25]. It is essential to establish a causal link between sequence variants and CHEK2 function. Little is known about the impact of missense mutations on protein function, although substitutions in the FHA domain and the kinase domain have been shown to abolish activity [22,26,27]. In this study, we screened the whole *CHEK2 *coding sequence for mutations in non-*BRCA *HBC families and a control population without any family history of breast cancer. Point mutations were evaluated by *in silico *analyses and an *in vitro *kinase activity test.

## Materials and methods

### Subjects

We recruited 507 cases with HBC risk through the oncogenetic consultation department at the Centre Jean Perrin (Clermont-Ferrand, France). This group consisted of 258 families with 3 breast cancers in the same familial branch with at least 2 cases related in the first degree, 237 families with 2 cases of breast cancer in the same branch with at least 1 breast cancer diagnosed before age 40 years or with bilateral breast cancer, and 12 families with 2 cases of breast cancer and at least 1 male breast cancer. One affected patient per HBC family was screened for variants in *CHEK2*. Cases with HBC linked to *BRCA1 *or *BRCA2 *mutations were excluded by direct sequencing of both genes and by multiplex ligation-dependent probe amplification of *BRCA1*. A control group recruited from the same region of France consisted of 513 female volunteers in good health and without any personal or family history of breast or gynecologic cancers at the time of the recruitment. All subjects signed informed consent agreements that were approved by the CCPPRB Regional Ethics Committee (Auvergne, France). To assess the relationship between *CHEK2 *variants and breast cancer risk, logistic regression was used to obtain odds ratios (as estimates of relative risk) and 95% confidence intervals [28].

### DNA extraction and sequencing

To identify variants in the *CHEK2 *gene, exons 2 to 14 were analyzed (exon 1 is noncoding, and exon 15, representing 89 bp of coding sequence, could not be analyzed for all the patients, owing to the presence of repeated sequences) in both patients and controls for the genomic sequence [GenBank:NG_008150.1] and for the cDNA sequence [GenBank:NM_007194.3] [29] DNA was extracted from 10 ml of peripheral blood collected on heparin/lithium using a Genomix blood DNA extraction kit according to the manufacturer's instructions (Talent srl, Trieste, Italy). Samples were resuspended with Tris-ethylenediaminetetraacetic acid (EDTA) (TE) (10 mM Tris, 1 mM EDTA, pH 8.0). Exons 2 to 10, including intron-exon boundaries, were amplified by using standard PCR techniques (conditions and primers available on request). Because of the multiple copies of *CHEK2 *pseudogenes, we used a nested PCR strategy, described previously by Sodha *et al*. [10], to specifically amplify exons 10 to 14 [30]. Sequence reactions were performed on PCR products purified by ExoSAP-IT (Affymetrix, Inc, Santa Clara, CA, USA) using BigDye v3 reagents (Applied Biosystems/Life Technologies, Foster City, CA, USA) (primers available on request), purified in Sephadex G-50 fine (G5080; Sigma-Aldrich, St Louis, MO, USA) and analyzed using a 3130xl capillary electrophoresis system (Applied Biosystems/Life Technologies). Alignment to the reference sequences was performed using SeqMan NGen software (DNASTAR, Inc, Madison, WI, USA).

### Bioinformatics studies

For each missense variant, prediction of the impact of the mutation on the protein was assessed by calculating the SIFT (Sorting Intolerant From Tolerant), Align-GVGD and PolyPhen-2 (Polymorphism Phenotyping v2) software tool scores [31-34]. Align-GVGD predictions and SIFT score were computed using the ortholog alignment of exons 2 to 14 of *CHEK2 *derived by using Alamut software (Interactive Biosoftware, Rouen, France) [32]. Included were human (*Homo sapiens*) [GenBank:NP_009125.1], chimpanzee (*Pan troglodytes*) [GenBank:XP_001172759.1], macaque (*Macaca*) [GenBank:XP_001101658.1], rat (*Rattus norvegicus*) [GenBank:NP_446129.1], mouse (*Mus musculus*) [GenBank:NP_057890.1], dog (*Canis lupus familiaris*) [GenBank:XP_543464.2], cow (*Bos taurus*) [GenBank:NP_001029703.1], chicken (*Gallus gallus*) [GenBank:XP_001232074.1], frog (*Xenopus tropicalis*) [GenBank:NP_001119996.1] and pufferfish (*Tetraodon nigroviridis*) [UniProtKB/TrEMBL:Q4TI84], all extracted from the Ensembl Compara database [35]. PolyPhen-2 score was calculated online using default settings and accession numbers [UniProtKB/Swiss-Prot:O96017] [36,37]. The potential impact on splicing was studied using SpliceSiteFinder, MaxEntScan and GeneSplicer prediction software [38-40].

### Plasmid constructs

The pDream2.1 cloning vector (GenScript USA Inc, Piscataway, NJ, USA) carrying the full-length human *CHEK2 *coding sequence tagged with an N-terminal FLAG extension under the control of the *LacZ *promoter for expression in prokaryotes was verified to contain the wild-type (WT) sequence. The Stratagene QuickChange II Site-Directed Mutagenesis Kit (Agilent Technologies, Inc, Santa Clara, CA, USA) was used to generate mutant constructs Ser39Phe, Pro85Arg, Arg117Gly, Arg145Trp, Glu161Del, Arg180His, Lys224Glu, Lys244Arg, Met367fsX15, Tyr390Ser and Thr476Met, with the corresponding primers (available on request) in accordance with the manufacturer's recommendations. All constructs were confirmed by sequencing of the entire coding region of the gene (primers available on request).

### Expression and extraction of recombinant CHEK2 protein

*Escherichia coli *strain BL21 was transformed with pDream plasmids(GenScript USA Inc) encoding WT or mutated Flag-*CHEK2*. Cultures were grown at 37°C in Luria Broth media containing 100 μg/ml ampicillin until absorbance at 600 nm reached 0.6 before isopropyl β-D-1-thiogalactopyranoside was added to a final concentration of 0.5 mM and incubated for 3 hours. Extraction of total bacterial proteins was performed as described previously [26].

### Kinase activity of CHEK2 recombinant proteins

Omnia kinase assay buffer (18 μl; Invitrogen/Life Technologies, Carlsbad, CA, USA) containing 10 μM Sox substrate peptide, 1 mM ATP, 0.2 mM dithiothreitol and 2 μl of 10 × Omnia buffer was incubated for 5 minutes at room temperature and aliquoted to a 96-well plate to ensure equal amounts of the chemosensor. For each assay reaction, 1.5 μg of total bacterial protein from induced cultures containing WT or mutated Flag-CHEK2 or from untransformed *E*. *coli*, were then added and mixed gently. CHEK2 protein was added at the moment of the fluorescence acquisition, allowing us to follow the kinetics of substrate phosphorylation. CHEK2 kinase activity was monitored with excitation at 360 nm and emission at 485 nm. Fluorescence was detected using an Infinite 200 PRO plate reader (Tecan Group Ltd, Männedorf, Switzerland) for 60 minutes at room temperature. For each mutation, an average of six wells and three independent experiments were conducted. Each curve was normalized by linear regression using the slope of the corresponding nontransformed bacterial protein extract curve. Thus the slope of the resulting curves represents the ability of CHEK2 recombinant protein to phosphorylate the substrate (see Additional file 1).

## Results

### *CHEK2 *mutations contribute to hereditary breast cancer

**Figure 1:**
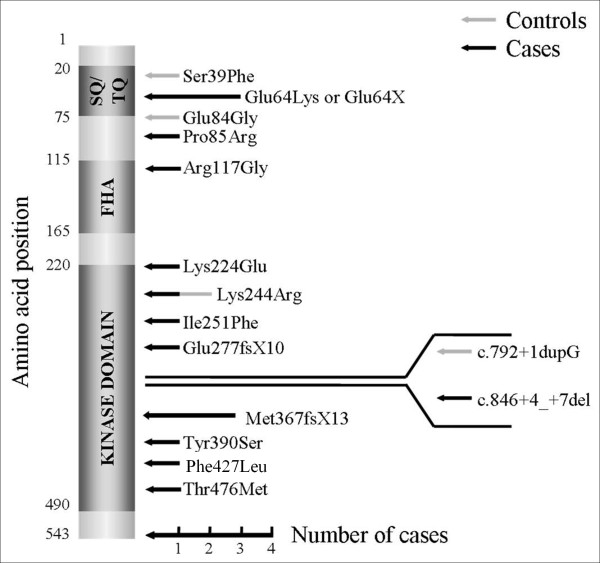
**Position of *CHEK2 *mutations found in French non-BRCA HBC and control populations**. FHA = Forkhead-associated domain; SQ/TQ = SQ/TQ cluster domain.

To evaluate the contribution of *CHEK2 *mutations to HBC, we sequenced the coding sequence of the gene, including intron-exon boundaries. We observed 13 different variants in 16 of 507 cases and 4 different variants in 4 of 513 controls (Table 1). In the case population, there were eight different novel missense mutations and one previously described in osteosarcomas [41], as well as one nonsense mutation, one novel frame shift mutation, one splice donor mutation and three patients (0.59%) with the c.1100delC (Met367fsX13) mutation (Figure 1). No mutation hotspots were observed (Figure 1). Mutations among controls included three missense mutations and one affecting a splice donor site. To the best of our knowledge, we are the first to report all mutations found in the control population. The missense mutation Lys244Arg was found in both cases and controls. The mutation frequency was higher for the cases (16 of 1,014 vs 4 of 1,026; *P *= 0.0065) (Table 2). The OR of *CHEK2 *mutation carriers was 4.15 (95% CI = 1.38 to 12.50), suggesting that *CHEK2 *contributes to hereditary risk of breast cancer.

**Table 1:** *CHEK2 *mutations identified in French women with hereditary breast cancer and a control group of unaffected women

Mutation	Amino acid change	Exon	Protein domain	Splicing effect	Align-GVGD class	SIFT median sequence conservation score	SIFT prediction	PolyPhen-2 prediction	Proposed diagnosis
Case population (*n *= 13)									
190G > A [49] (two cases)	Glu64Lys	2	SQ/TQ	-	C15	2.23	Tolerated	Benign	Probably benign
190G > T	Glu64X	2	SQ/TQ	-	-		-	-	Probably deleterious
254C > G [23,41]	Pro85Arg	2	-	-	C0	2.15	Tolerated	Probably damaging	Probably deleterious
349A > G [23]	Arg117Gly	3	FHA	-	C65	2.07	Deleterious	Probably damaging	Probably deleterious
670A > G	Lys224Glu	5	Kinase	-	C0	2.05	Tolerated	Benign	Probably benign
731A > G	Lys244Arg	6	Kinase	-	C0	2.04	Tolerated	Benign	Probably benign
751A > T [23]	Ile251Phe	6	Kinase	-	C0	2.04	Tolerated	Probably damaging	Probably deleterious
846+4_846+7del	-	7	-	Possibly skip exon 7	-	-	-	-	Probably deleterious
1100del (three cases)	Thr367MetfsX15	11	Kinase	-	-	-	-	-	Probably deleterious
1169A > C	Tyr390Ser	11	Kinase	-	C0	2.04	Deleterious	Probably damaging	Probably deleterious
1281C > A	Phe427Leu	12	Kinase	-	C0	2.04	Deleterious	Probably damaging	Probably deleterious
1427C > T [23]	Thr476Met	13	Kinase	-	C0	2.05	Deleterious	Probably damaging	Probably deleterious
Control population (*n *= 4)									
116C > T	Ser39Phe	2	SQ/TQ	-	C65	3.10	Deleterious	Benign	Probably deleterious
251A > G	Glu84Gly	2	-	-	C65	2.17	Tolerated	Probably damaging	Probably deleterious
731A > G	Lys244Arg	6	Kinase	-	C0	2.04	Tolerated	Benign	Probably benign
792+1dup	-	6	-	Probably skip exon 6	-	-	-	-	Probably deleterious
Additional mutations tested for kinase function [27]									
410G > A	Arg137Gln	3	FHA	-	C0	2.22	Tolerated	Benign	Probably benign
433C > T	Arg145Trp	3	FHA	-	C65	2.05	Tolerated	Probably damaging	Probably deleterious
481_483del	Glu161del	4	FHA	-	-	-	-	-	Probably deleterious
539G > A	Arg180His	4	FHA	-	C0	2.04	Tolerated	Probably damaging	Probably deleterious

Align-GVGD, SIFT (Sorting Intolerant From Tolerant) and PolyPhen-2 (Polymorphism Phenotyping v2) software tool scores and splicing effect were calculated. The possible impact of the different mutations on CHEK2 function is proposed. FHA = Forkhead-associated domain.

**Table 2:** *CHEK2 *mutations were more frequent in cases than in controls

*CHEK2 *mutation type	Controls (*n *= 1,026)^a^	Allelic frequency in controls	Cases (*n *= 1,014)^a^	Allelic frequency in cases	P value	OR	95% CI
Any *CHEK2 *mutation	4	0.39%	16	1.58%	0.0065	4.15	(1.38 to 12.50)
*CHEK2 *deleterious mutations	3	0.29%	15	1.48%	0.0042	5.18	(1.49 to 18.00)

^a^*n *indicates the number of alleles.

### Bioinformatics study

Canonical splice donor and acceptor sites were evaluated using SpliceSiteFinder, MaxEntScan and GeneSplicer. All three programs provided consistent information that the two mutations affecting splice donor sites abrogate splicing of the exons concerned (Table 1). We thus considered these mutations to be deleterious. Because the effect of an amino substitution can be difficult to assess, a combination of three different *in silico *analyses (Align-GVGD class, SIFT prediction and PolyPhen-2 prediction) was used. For each missense variation, we compiled these three scores to propose a diagnosis. Missense variants were considered probably deleterious if at least one deleterious score was obtained and probably benign if three benign scores were obtained. Class above C35 was considered the threshold for deleterious variants in Align-GVGD.

Substitutions with a SIFT score less than 0.05 are predicted to be deleterious. A SIFT median sequence conservation score cutoff of 3.25 was used to measure the diversity of the sequences used for prediction, and a score greater than 3.25 could indicate that the prediction was based on closely related sequences. This would result in a low confidence score if the variant were considered deleterious. No SIFT median sequence conservation score reached this cutoff, indicating that the aligned sequences were diverse enough for confident prediction of substitutions that should affect protein function. One mutation, Lys244Arg, present in both cases and controls, was not considered to be potentially deleterious on the basis of the results of any of the algorithms used, suggesting it is a rare but benign variant. All other missense variants were considered potentially damaging on the basis of at least one measure.

### Effect of *CHEK2 *mutations on kinase activity

**Figure 2:**
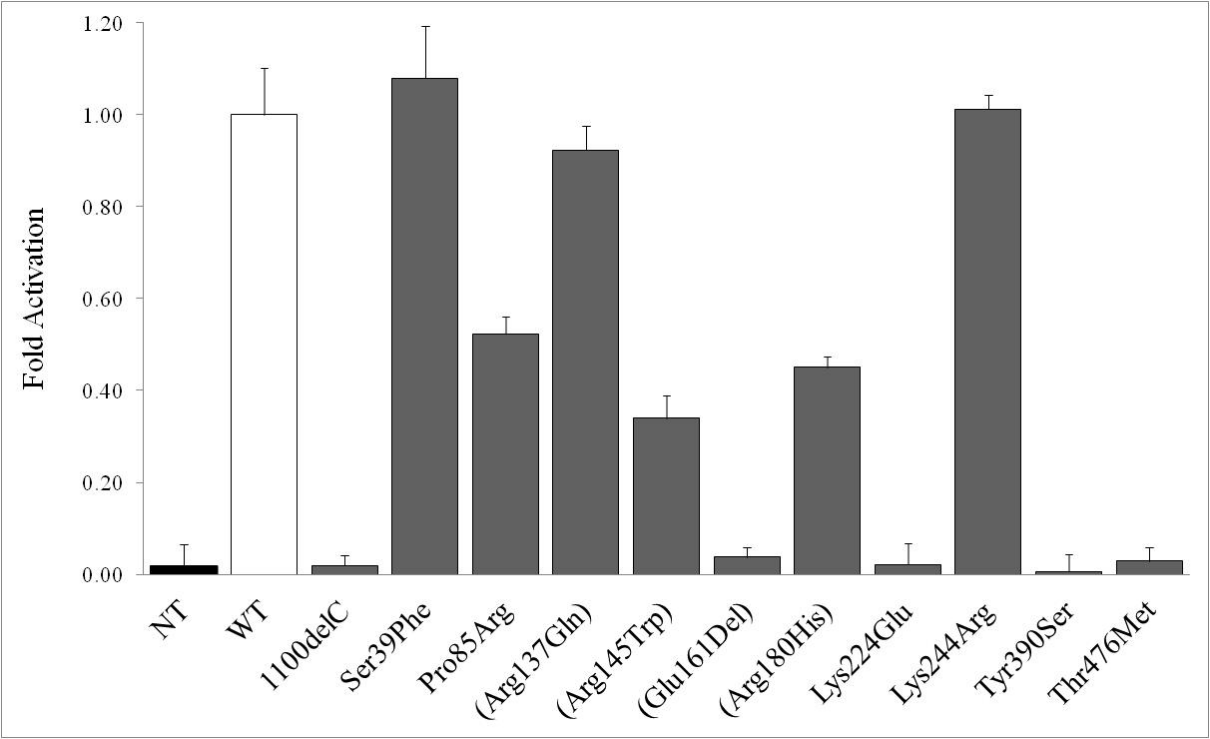
**Kinase activity of recombinant Flag-CHEK2 protein**. Total protein extract (1.5 μg) was tested for the ability to phosphorylate a fluorescent substrate. The slope of the resulting curve represents Flag-CHEK2 kinase activity. The slope of the wild-type (WT) Flag-CHEK2 kinase activity curve was normalized to 1. Nontransformed protein extracts (NT) and mutant c.1100delC served as controls. Each point represents an average of five measurements performed in triplicate.

To evaluate whether missense variants inhibit the function of the CHEK2 protein, an *in vitro *kinase activity test based on a CHEK2-specific substrate peptide carrying a C-terminal SOX was developed [42]. Overexpression of recombinant CHEK2 at high levels in bacteria is associated with CHEK2 autophosphorylation and activation in the absence of DNA damage [13]. This property was used to obtain recombinant activated CHEK2. Upon the phosphorylation of the SOX-specific substrate by CHEK2, the presence of the chemosensor SOX results in an increase in fluorescence at 485 nm. Activity was detected for the WT protein but not for proteins extracted from nontransformed bacteria or recombinant CHEK2 protein carrying c.1100delC (Figure 2). Only missense variants were tested for kinase activity. c.190G > T (Glu64X), c.825_826del, c.846+4_+7del and c.792+1dup were considered deleterious without further analysis. Four mutations tested for kinase activity *in vitro *by Sodha *et al*. [27] were included to validate the assay.

Three different classes of kinase activity were observed: WT-like, intermediate and null (Figure 2). Mutations Ser39Phe, Arg145Trp and Arg137Gln exhibited WT-like kinase activity, suggesting that these mutations do not affect the ability of recombinant CHEK2 to recognize, bind and phosphorylate its substrate (Figure 2). The mutants Pro85Arg, Arg180His and Lys244Arg had significantly lower, but not null, kinase activity (Figure 2), which placed them in the intermediate class. The mutations Glu161Del, Lys224Glu, Thr476Met and Tyr380Ser did not have any kinase activity. Nine of the eleven mutations showed kinase activity consistent with the *in silico *analysis, demonstrating the good but incomplete correlation of those two approaches (Table 3).

**Table 3:** Relationship between *in silico *and *in vitro *results

Mutation	Domain	*In silico *conclusion	*In vitro *conclusion	Concordance
Ser39Phe	SQ/TQ	Probably deleterious	WT-like	No
Pro85Arg	Unknown function	Probably deleterious	Intermediate	Yes
Arg137Gln	FHA	Probably benign	WT-like	Yes
Arg145Trp	FHA	Probably deleterious	Intermediate	Yes
Glu161Del	FHA	Probably deleterious	Null	Yes
Arg180His	FHA	Probably deleterious	Intermediate	Yes
Lys 224Glu	Kinase core	Probably benign	Null	No
Lys244Arg	Kinase core	Probably benign	WT-like	Yes
Met367fsX13	Kinase core	Probably deleterious	Null	Yes
Tyr390Ser	Kinase core	Probably deleterious	Null	Yes
Thr476Met	Kinase core	Probably deleterious	Null	Yes

Both *in silico *and *in vitro *analyses suggested that the variant Lys244Arg, present in cases and controls, can be considered benign. This variant was thus removed from the pool of potentially deleterious *CHEK2 *variants that contribute to HBC. As a result, the mutation frequency was reduced to 1.48% for cases and 0.29% for controls (Table 2). This difference remained significant (*P *= 0.0042), and the OR associated with the presence of a deleterious mutation was increased to 5.18 (95% CI: 1.49 to 18.00).

## Discussion

We found strong evidence of an association between *CHEK2 *variants and HBC, with an OR of 5.18. Of 16 different mutations, 9 were unreferenced variants. This demonstrates that, in populations without founder mutations, an aggregate of rare variants makes *CHEK2 *an appreciable breast cancer risk gene.

The functional consequences of missense variants can be difficult to establish, and in estimating associated risks it is important to separate deleterious from neutral variants. We were unfortunately unable to complement the functional data presented here with a study of the cosegregation of these variants with cancer, because only the index case was available for analysis in the majority of families.

Missense variant Ser39Phe was predicted as probably deleterious by two of the three scores (SIFT and Align-GVGD), but exhibited WT-like kinase activity. This discordance may suggest that not all deleterious changes in the CHEK2 protein can be revealed by the *in vitro *kinase activity test, most notably for changes outside the catalytic domain. Changes affecting interactions with upstream activators such as ATM, for example, may not be detectable by our measure. In contrast, Lys224Glu was predicted to be a tolerable change by the three scores, but exhibited null kinase activity, demonstrating the complementarity of those two approaches.

Further functional tests, such as expression in eukaryotic cells, followed by measures of activation by DNA strand breaks, protein stability and interaction with cellular partners may be necessary to appreciate all effects of these mutations, especially for those where the *in silico *and *in vitro *conclusions differ. We thus retain this variant as potentially deleterious, unlike Lys244Arg, which was characterized as benign by all measures.

The association between the *CHEK2 *gene and breast cancer risk has been supported mainly by case-control studies of founder mutations such as 1100delC, I157T (frequent in northern and eastern Europe) or the Polish founder mutation IVS2+1 G > A (c.444+1G > A) [19,20,24,43,44]. In our population, only one of these founder mutations was observed, accounting for one-third of deleterious mutations. Analysis of the entire coding sequence was necessary to capture the majority of the different mutations present. This might be the case for other populations where the frequency of the *CHEK2 *founder mutations is low.

In Table 4, to give an overview of *CHEK2 *contribution to breast cancer, we summarize the results of 36 different case-control studies from different countries where the presence of variants was assessed by allele-specific sequencing or DNA sequencing of the entire gene. The ORs of breast cancer from the different studies of c.1100delC are similar, regardless of the selection of cases, with a combined OR of 2.77. We also found comparable results for the other protein-truncating mutation c.444+1G > A, which is less frequent but has an OR similar to that for c.1100delC. No positive association with HBC was observed, possible due to the very low frequency of the variant in both cases and controls. The frequent variant I157T was associated with lower ORs than null mutations. Although this variant has been associated with breast cancer risk in early-onset or unselected cases, in our study it did not exhibit a significant association with HBC. Although the frequency of these deleterious mutations was different among populations, the ORs associated with breast cancer were consistent for the two null mutations and lower for the missense mutation. These data were collected using allele-specific sequencing, suggesting that testing for *CHEK2 *founder mutations is cost-effective in some populations because the variants are sufficiently common and the test is relatively inexpensive. Consequently, however, these techniques exclude mutations present elsewhere in the gene.

**Table 4:** Odds ratio for breast cancer among women with *CHEK2 *variants

Variants	References	Geographic populations	Case populations	Mutation frequency in controls (*n*/total)	Mutation frequency in cases (*n*/total)	OR (95% (CI)
c.1100delC		Mixed populations	All studies	0.33% (559/166,596)	0.90% (861/94,076)	2.77 (2.49 to 3.08)**
	[19,22,25,45,47,50-59]	AJ, AUS, B, CDN, CZ, DK, E, FIN, G, IRL, KP, NL, S, UK, USA	UBC	0.31% (2,502/80,168)	0.80% (464/58,290)	2.56 (2.19 to 2.99)**
	[3,19,22,24,25,50,54,60-64]	B, CDN, CZ, D, FIN, NL, S, UK, USA	HBC	0.39% (111/28,402)	1.27% (215/17,000)	3.29 (2.61 to 4.14)**
	[23,54,65]	D, USA, AUS, CDN	EOBC	0.14% (14/9,846)	0.46% (21/4,588)	2.77 (1.23 to 6.26)**
	[18,53,60,66,67]	AJ, DK, G, FIN, NL, RUS, UK, USA	BBC	0.43% (130/29,936)	1.35% (142/10,496)	3.17 (2.49 to 4.03)**
c.444+1G > A (IVS2+1G > A)		Mixed populations	All studies	0.19% (82/42,266)	0.66% (217/33,142)	3.45 (2.67 to 4.46)**
	[68,69]	PL	EOBC	0.19% (49/25,426)	0.59% (91/15,338)	3.10 (2.19 to 4.39)**
	[24,43]	D, BY	HBC	0.12% (3/2,586)	0.26% (4/1,536)	2.25 (0.5 to 10.08) (NS)
	[43,59,70]	D, BY, PL	UBC	0.20% (28/14,254)	0.61% (113/18,604)	3.12 (2.06 to 4.72)**
I157T		Mixed populations	All studies	2.14% (1,031/48,268)	3.11% (1062/34,128)	1.56 (1.43 to 1.70)**
	[22,23,65,68]	PL, USA, AUS, CDN	EOBC	1.46% (270/18,432)	1.99% (215/10,780)	1.59 (1.32 to 1.91)**
	[20,22,24,43,71]	D, BY, FIN, NL, USA	HBC	1.77% (116/6,544)	1.51% (47/3,118)	0.89 (0.60 to 1.20) (NS)
	[20,22,25,43,44,59,70]	By, CZ, D, FIN, PL, USA	UBC	2.04% (644/31,600)	2.95% (807/27,346)	1.48 (1.33 to 1.65)**
Whole-gene studies	[22]	USA	UBC	0.57% (24/4,210)	0.50% (4/800)	0.88 (0.3 to 2.55) (NS)
	[24]	D	HBC	0.50% (18/3,630)	3.00% (31/1,032)	6.38 (3.54 to 11.5)**
	[25]	CZ	HBC	1.39% (19/1,366)	2.01% (27/1,346)	1.46 (0.80 to 2.65) (NS)
		F	HBC	0.39% (4/1,026)	1.58% (16/1,014)	4.15 (1.38 to 12.50)**
	[23]	USA, CDN, AUS	EOBC	1.84% (40/2,178)	4.91% (6/2,484)	2.76 (1.65 to 4.60)**

BBC = bilateral breast cancer; EOBC = early-onset breast cancer; HBC = hereditary breast cancer not related to a *BRCA *mutation; UBC = unselected breast cancer; AJ = Ashkenazi Jewish; AUS = Australia; B = Belgium; BY = Belarus; CDN = Canada; CZ = Czech Republic; D = Germany; DK = Denmark; E = Spain; F = France; FIN = Finland; IRL = Ireland; KP = South Korea; NL = The Netherlands; PL = Poland; RUS = Russia; S = Sweden; UK = United Kingdom; USA = United States of America; NS = nonsignificant (*P *< 0.05); **P < 0.0001. Participant numbers may vary from original publications because of exclusion of study subgroups.

Because the c.1100delC allele does not seem to be present in southern Europeans or in most non-Caucasian populations [45-47], other research groups have used full-gene sequencing to determine whether other variants contribute to breast cancer risk. There is a positive association between *CHEK2 *variants and HBC in the Australian, Canadian, North American, German and now French, but not Czech Republic, populations [22-25]. This suggests that *CHEK2 *analysis in populations where the common founder mutations are rare requires screening of the entire sequence.

Narod's [48] recent review supports the view that testing non-*BRCA *HBC families for mutations in *CHEK2 *can provide useful information to evaluate the risk of breast cancer and suggests that the relatively high cost of sequencing makes only the targeted search of frequent mutations cost-effective. In certain populations, one or a few mutations do indeed capture the majority of *CHEK2 *variants associated with cancer risk. In most regions, however, this allele-specific approach is inadequate and a full-resequencing strategy should be considered. The rapidly falling cost of resequencing, as well as alternate techniques, should make this possible.

## Conclusions

The usefulness of the information gained from genetic analysis of *CHEK2 *is currently a matter of debate. As we have discussed, the risk of breast cancer for a woman with a null mutation in this gene is increased two- to fivefold. Increased breast surveillance may be proposed for carriers, but when counseling a family with many breast cancer cases, only some of whom carry the *CHEK2 *mutation, it is unclear what advice may be given to noncarriers. Collecting research information on *CHEK2 *mutations, however, serves to advance our understanding of the contribution of this gene to hereditary cancer risk.

## Abbreviations

HBC: hereditary breast cancer; FHA: Forkhead-associated; WT: wild type.

## Competing interests

The authors declare that they have no competing interests.

## Authors' contributions

AD contributed to the sequencing of *CHEK2*, designed and performed the kinase activity, participated in the *in silico *analyses and drafted the manuscript. YB provided expert technical advice, and helped to draft the manuscript. NU designed the study, participated in the sequencing of *CHEK2 *and provided expertise for the *in silico *analyses. YJB supervised the study and helped to draft the manuscript. All authors read and approved the final manuscript.

## Web resources

The URLs for the accession numbers and data presented herein are as follows:

Entrez gene database: http://www.ncbi.nlm.nih.gov/gene (for *CHEK2 *sequencing)

Ensembl Compara database: http://www.ensembl.org/info/docs/compara/index.html

UniProtKB/Swiss-Prot database: http://www.uniprot.org/uniprot

## Supplementary Material

Additional file 1 **Supplementary data: control kinase activity of recombinant CHEK2 protein**. Total protein extract (1.5 μg) was added to the substrate peptide. Fluorescence was measured at 485 nm for 1 hour. Wild type (WT), nontransformed protein extracts (NT) and mutant c.1100delC served as controls, and kinase activity of bacterially expressed mutants are sorted by domain. Each point on the curve represents an average of six measurements repeated in triplicate. Click here for file
