# Supplementary material for: CHEK2 contribution to hereditary breast cancer in non-BRCA families
Source: Breast Cancer Res. 2011 Nov 24;13(6):R119. doi: 10.1186/bcr3062 (PMC3326561; doi:10.1186/bcr3062)

Supplementary Data

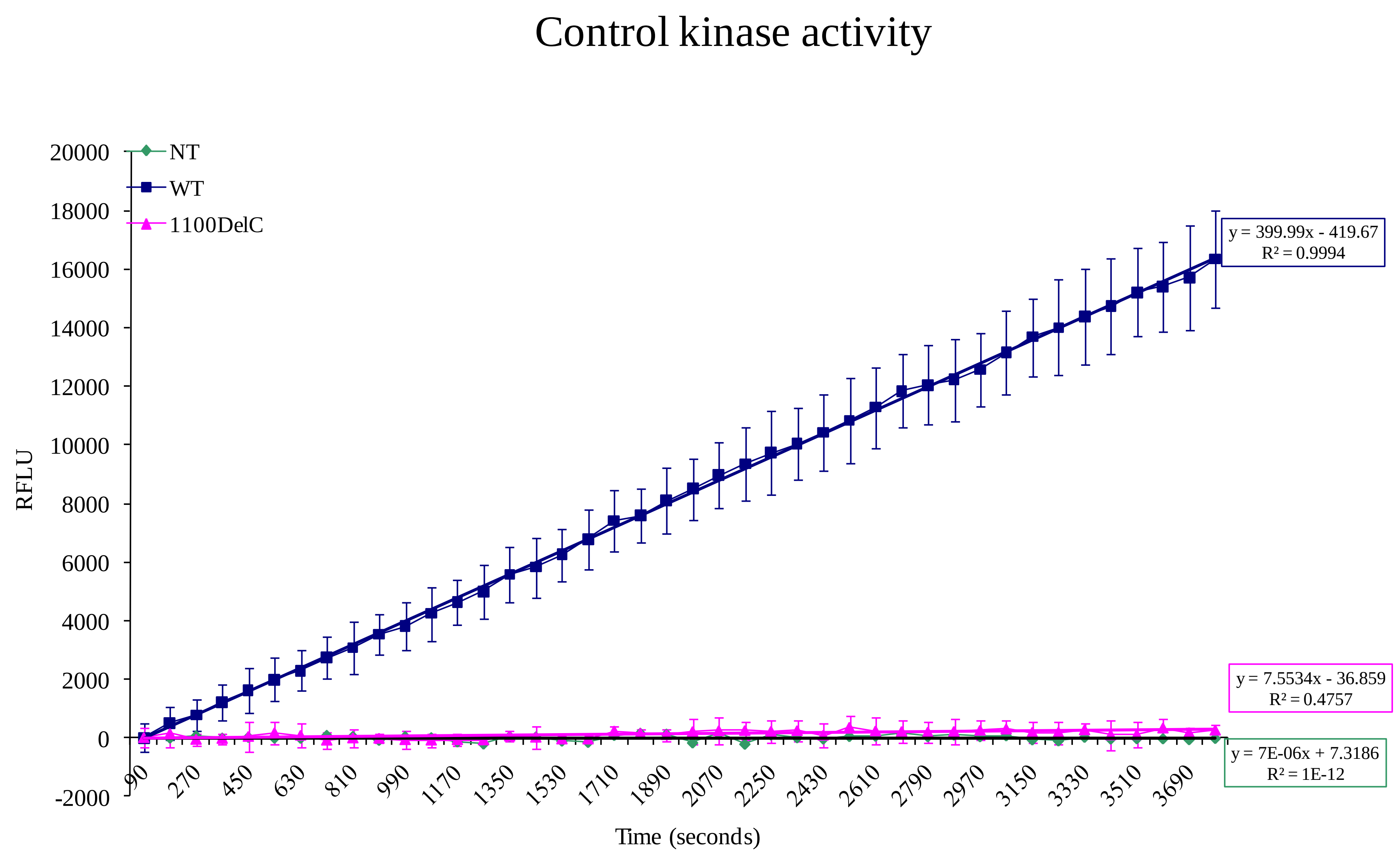

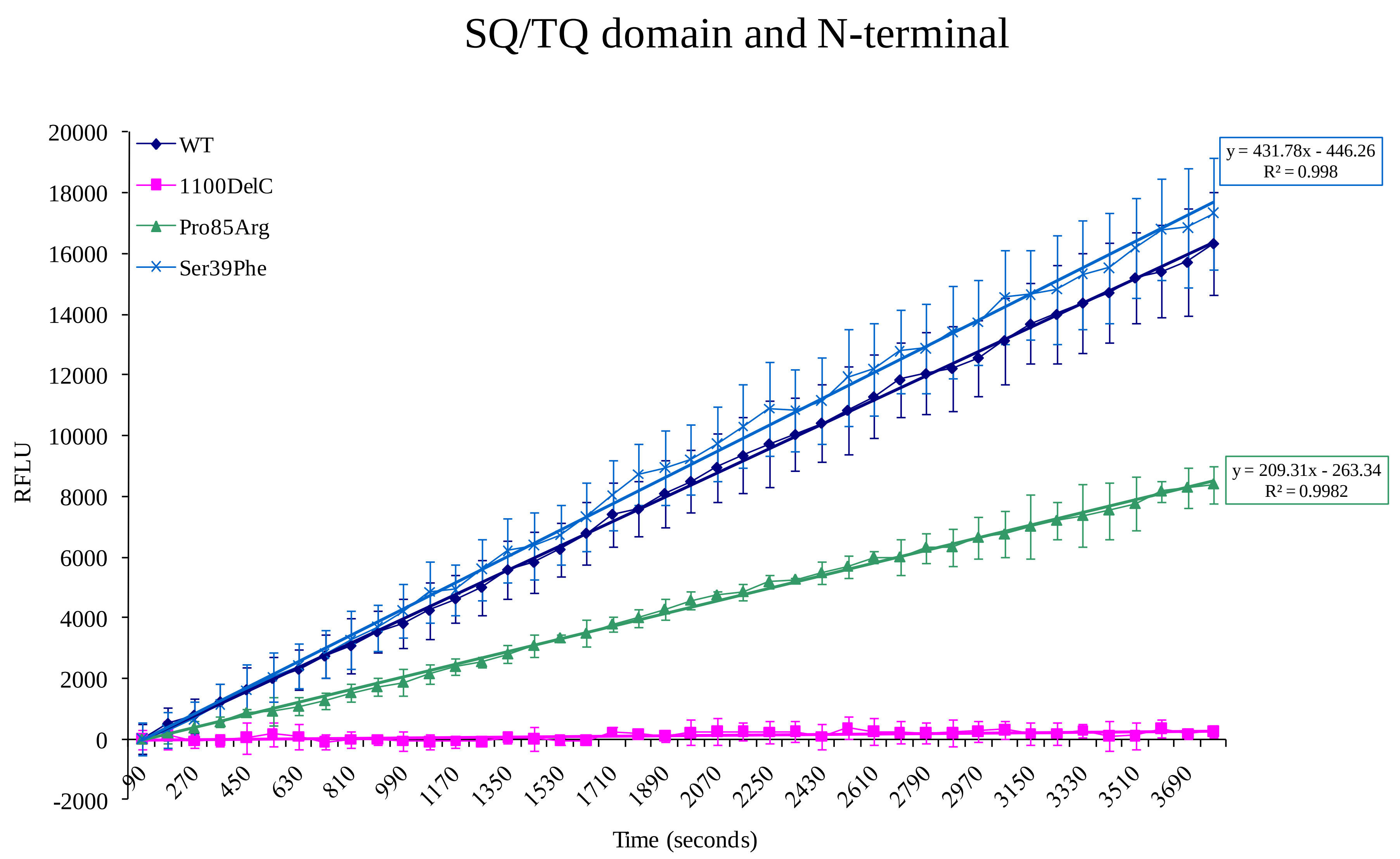

FHA domain

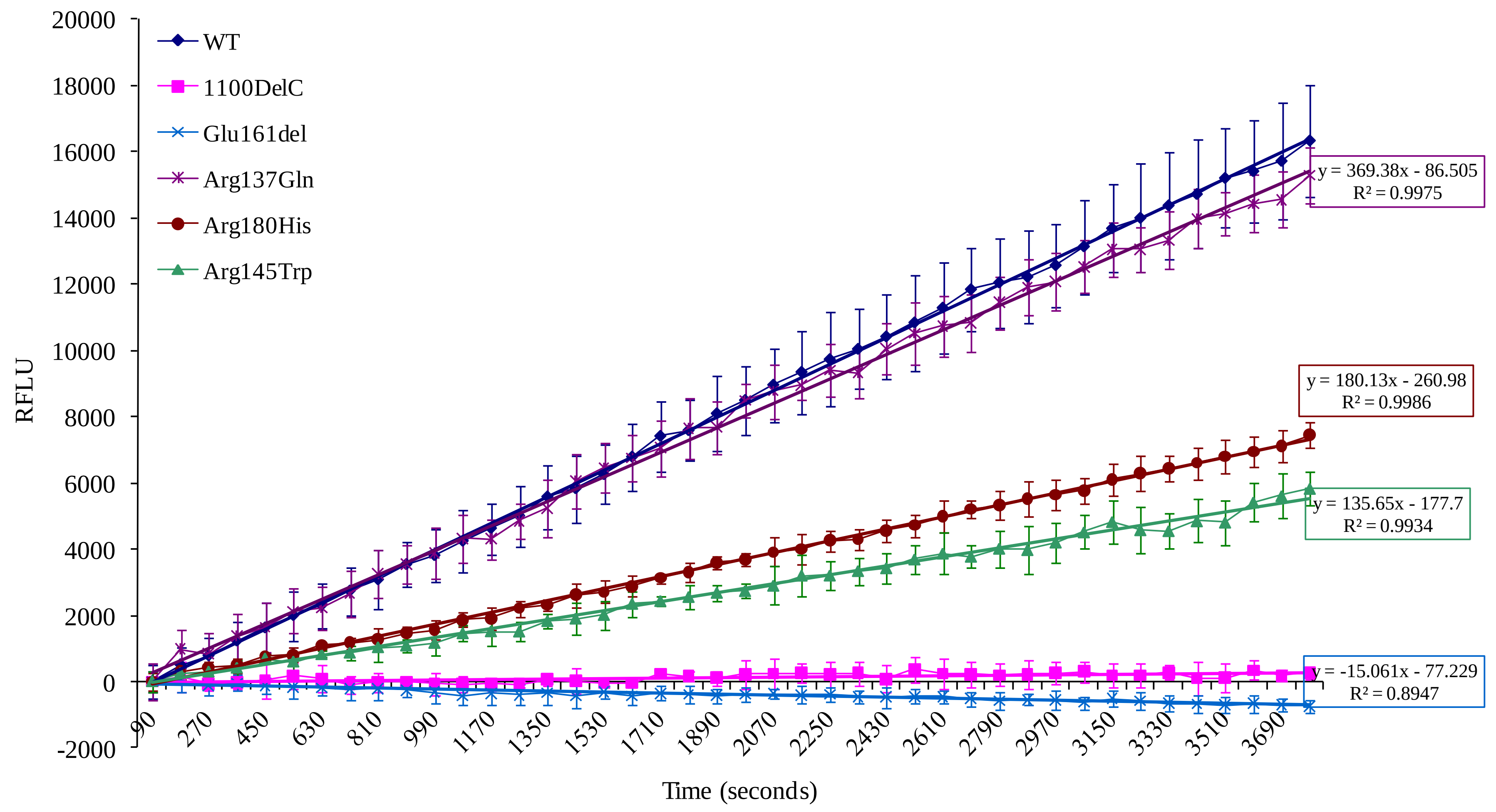

Kinase domain

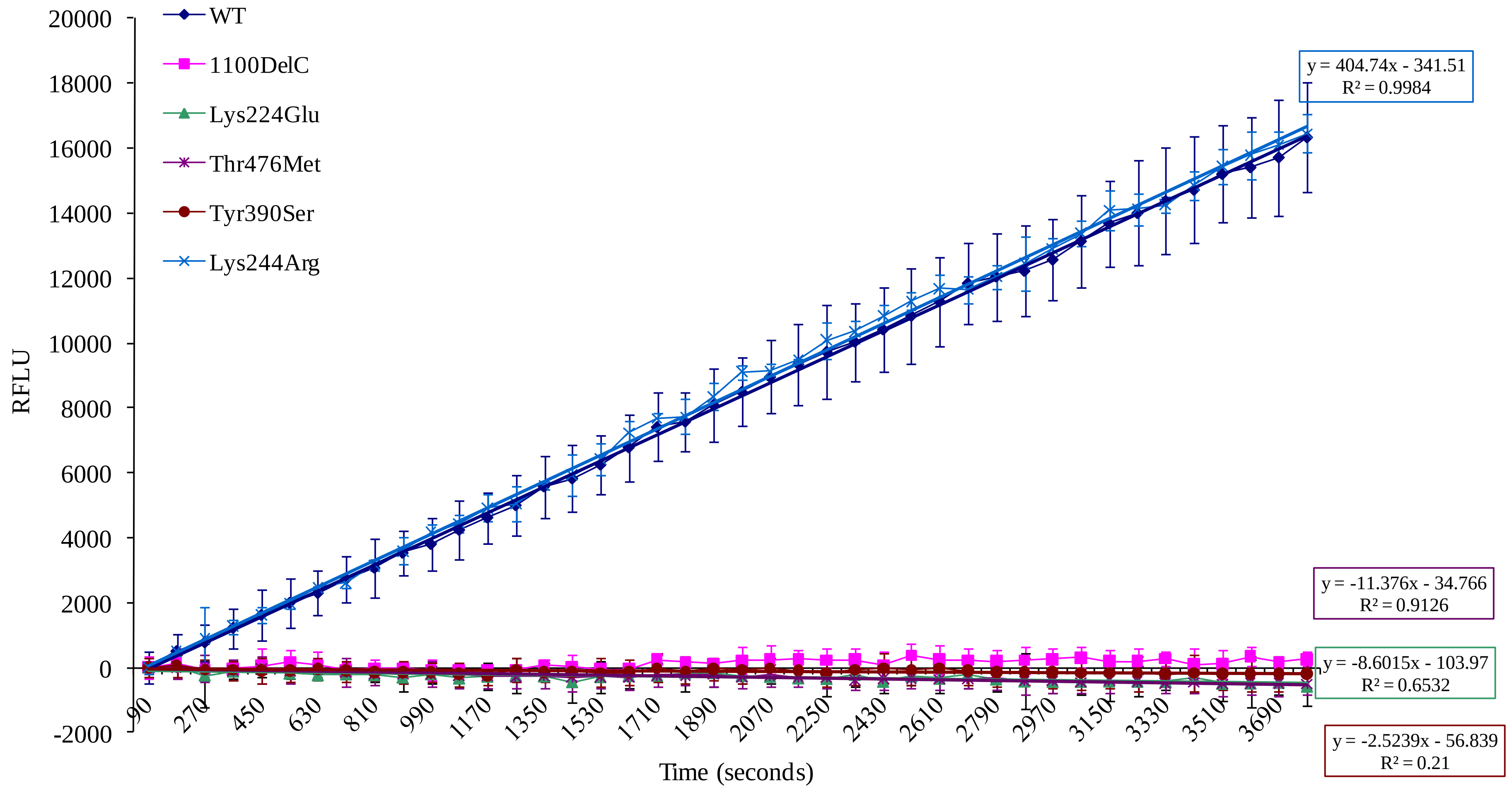

Supplement: Additional file 1 — Supplementary data: control kinase activity of recombinant CHEK2 protein. Total protein extract (1.5 μg) was added to the substrate peptide. Fluorescence was measured at 485 nm for 1 hour. Wild type (WT), nontransformed protein extracts (NT) and mutant c.1100delC served as controls, and kinase activity of bacterially expressed mutants are sorted by domain. Each point on the curve represents an average of six measurements repeated in triplicate. [file bcr3062-S1.PDF]
